# Bioactive Molecules of Tea as Potential Inhibitors for RNA-Dependent RNA Polymerase of SARS-CoV-2

**DOI:** 10.3389/fmed.2021.684020

**Published:** 2021-05-31

**Authors:** Vijay Kumar Bhardwaj, Rahul Singh, Jatin Sharma, Vidya Rajendran, Rituraj Purohit, Sanjay Kumar

**Affiliations:** ^1^Structural Bioinformatics Lab, CSIR-Institute of Himalayan Bioresource Technology (CSIR-IHBT), Palampur, India; ^2^Biotechnology Division, CSIR-IHBT, Palampur, India; ^3^Academy of Scientific and Innovative Research (AcSIR), Ghaziabad, India

**Keywords:** RNA-RdRp, bioactive molecules, SARS-CoV-2, tea, COVID-19

## Abstract

The coronavirus disease (COVID-19), a worldwide pandemic, is caused by the severe acute respiratory syndrome-corona virus-2 (SARS-CoV-2). At this moment in time, there are no specific therapeutics available to combat COVID-19. Drug repurposing and identification of naturally available bioactive molecules to target SARS-CoV-2 are among the key strategies to tackle the notorious virus. The enzyme RNA-dependent RNA polymerase (RdRp) performs a pivotal role in replicating the virus. RdRp is a prime target for Remdesivir and other nucleotides analog-based antiviral drugs. In this study, we showed three bioactive molecules from tea (epicatechin-3,5-di-O-gallate, epigallocatechin-3,5-di-O-gallate, and epigallocatechin-3,4-di-O-gallate) that showed better interaction with critical residues present at the catalytic center and the NTP entry channel of RdRp than antiviral drugs Remdesivir and Favipiravir. Our computational approach to identify these molecules included molecular docking studies, followed by robust molecular dynamics simulations. All the three molecules are readily available in tea and could be made accessible along with other medications to treat COVID-19 patients. However, these results require validation by further *in vitro* and *in vivo* studies.

**Graphical Abstract:**
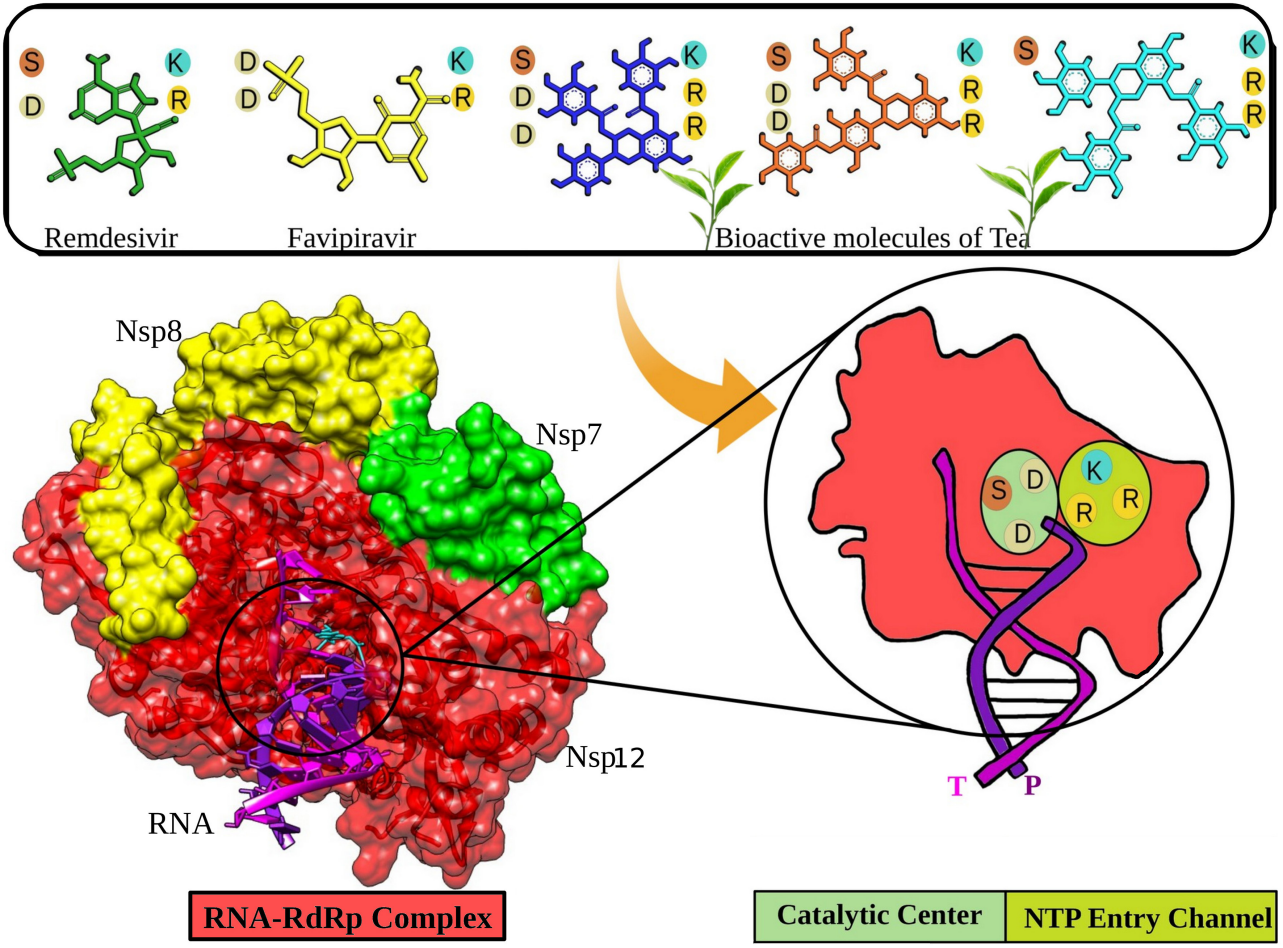


## Introduction

Recently, a major threat to humanity has emerged in the form of a novel coronavirus (CoV), causing a disease that is regarded as coronavirus disease 2019 (COVID-19) (1, 2). Taxonomically, this virus hails to the Coronaviridae family, which contains the enveloped positive-sense RNA virus of four major groups, alpha, beta, gamma, and delta (3, 4). Among these, Severe Acute Respiratory Syndrome (SARS) CoV and Middle East Respiratory Syndrome (MERS) CoV from the beta group are highly pathogenic to humans and develop symptoms like common cold, fever, and respiratory problems (5, 6). Previous outbreaks of the CoV in humans were reported in 2002 and 2012, which involved the SARS and MERS CoV, respectively (7, 8). Due to the absence of a specific treatment protocol, they had to be controlled via several public health measures (4, 9). The COVID-19 disease is provoked by a new CoV, named SARS-CoV-2. As compared to the other CoVs, SARS-CoV-2 has an uplifted human-to-human transmission rate, which gives a rationale for its extensive spread (10, 11).

The non-structural protein 12 (nsp12), also called the RNA-dependent RNA polymerase (RdRp), performs a significant function in the replication and transcription cycles of SARS-CoV-2 by catalyzing the synthesis of the viral RNA, making it one of the most critical targets for viral inhibition (1, 12). The nsp12 is likely to be assisted by cofactors like the nsp7 and nsp8 (13). The RdRp structure comprises a polymerase domain ranging from residue S367 to F920 that resembles a cupped “right hand” and a nidovirus-unique N-terminal extension domain ranging from residues D60 to R249, also called the NiRAN domain (14, 15). The two domains interact via the interface domain ranging from residues A250 to R365 (12). It also comprises the fingers subdomain (residues L366-A581 and K621-G679), the palm domain (residues T582-P620 and residues T680-Q815), and the thumb domain (residues H816-E920) (12, 14). There is an N-terminal β-hairpin (residues D29 to K50) between the palm subdomain and the NiRAN domain, which assists in the overall stabilization of the structure (12). The region from residue A4 to R118 comprises two helices and five antiparallel β-strands (9, 12). Another β-strand is present in the region between residues N215 and D218. It interacts with a strand in the region V96 to A100, thereby providing stability to the conformation by forming a compact and firm β-barrel architecture (12). The RdRp mediates a template-directed RNA synthesis part of the viral life cycle where the template entry, the nucleoside triphosphate (NTP) entrance, and the nascent strand exit pathway converge into a central cavity, which is all positively charged (12, 16). The NTP entry channel is demarcated via hydrophilic motif F having K545, R553, and R555 residues (12). The RNA template enters the active site from a channel between motifs F and G, where motif E and the thumb subdomain hold the template strand. The active site is mainly constituted of motifs A and C, held up by motifs B and D (9).

The worldwide spread of SARS-CoV-2 and the rising statistics emphasize the importance of identifying drug candidates, which can act as potent antivirals to control the growing pandemic. RdRp is a promising target for inhibition, firstly, due to its critical involvement in the viral life cycle; secondly, it conserved the nature of its structure and sequence across several RNA viruses; and lastly, due to the missing homologs in the host (4, 12). Nucleotide analogs (NAs) include Remdesivir (adenosine analog), which has already proven to be effective against several viral diseases and has also been reported to inhibit SARS-CoV-2 by controlling its proliferation (17, 18). NAs upon entry tend to acquire an active 5′-triphosphate, which challenges the endogenous nucleotides to get incorporated in the viral RNA by acting as an alternate substrate for the RdRp (1). Remdesivir also works similarly by benefiting from the low fidelity of the RdRp, thereby preventing viral proliferation by chain termination (19, 20). Analysis of the Remdesivir-mediated inhibition state of the virus illustrates conformational changes in the residues D760, D761, and D618. This allows the phosphate group of the inhibitor to interact with allosteric residue R555 (12).

In this study, a dataset of bioactive molecules of tea were screened and compared to Remdesivir and Favipiravir for their inhibitory potential against the RdRp of SARS-CoV-2. Tea is consumed by more than half of the world's population. The proof underpinning the health benefits of tea is rapidly growing with each new research published in the scientific literature. A plethora of studies have reported advantageous effects of habitual tea consumption against several types of cancers, cardiovascular diseases, diabetes, and arthritis (21). Apart from it, bioactive tea molecules are promising compounds in manifesting antiviral activities. They exhibit antiviral activity against a broad spectrum of human viruses, including HIV, herpes simplex virus, influenza, hepatitis B, and hepatitis C (22, 23). These compounds are even effective against Zika, Chikungunya, and Dengue viruses (23). Recent computational and experimental investigations documented the potent antiviral activities of bioactive tea molecules against multiple vital proteins, including the main protease (Mpro), non-structural protein 15 (Nsp15), spike, and RdRp of SARS-CoV-2 (24–28). The main objectives of the study were to analyze the interaction pattern and binding affinity of selected bioactive molecules and FDA-approved antiviral drugs with the active pocket of SARS-CoV-2 RdRp and, furthermore, to rank and suggest topmost molecules on the basis of their potential to hinder the replication process of SARS-CoV-2.

## Materials and Methods

### Datasets

The crystallographic RdRp-RNA structure complex (PDB Id: 7BV2) was obtained from the protein data bank with a resolution of 2.50 Å (29). The chain A (nsp12) contains 951 amino acids. The protein complex also constitutes of primer and template RNA strands of length 20 and 30 nucleotides, respectively. A set of bioactive molecules of Tea (24) was prepared for molecular docking and MD simulation studies. The structures of Remdesivir and Favipiravir in their active forms were obtained from PubChem (30).

### Molecular Docking

A set of bioactive molecules from tea along with Remdesivir and Favipiravir was docked into the active site of RdRp of SARS-CoV-2. Discovery Studio's CDOCKER algorithm was utilized to carry out molecular docking. CDOCKER is CHARMm-based semiflexible docking engine (31). The resilient conformation section grabbed by ligand molecules searched employing high-temperature kinetics. The optimization at the binding site of each conformation is achieved by using the simulated annealing method to obtain reliable docking outcomes. The CDOCKER parameters were kept on default. The number of starting random conformations and the number of rotated ligand orientations to refine for each of the conformations for 1,000 dynamics steps were set to 10. Moreover, for annealing refinement, the number of heating steps was 2000, while the number of cooling sets was set to 5,000. The distance to consider Pi-cation, Pi-Pi, and Pi-alkyl interactions was set to 5, 6, and 5.5 Å, respectively. A radius of 8.0 Å was assigned centering the ligand in the active site that contains all the active residues participating in the binding of the ligand to the RdRp–RNA SARS-CoV-2 protein. The 3D structures of all the bioactive molecules were prepared using the Discovery Studio package (32). Furthermore, for energy minimization, we have used CHARMm force field and DFT protocols (33). The built molecules were then read in Discovery Studio.

### Molecular Dynamics Simulations

A 100-ns MD simulation was performed for all the selected complexes using the GROMACS 4.6.7 suite (34). A large protein size of RdRp (951 amino acids) along with RNA increases the complexity of all atomic simulation setups by several folds. The protein topology for the protein complexes has been derived from the CHARMM27 force field, while ligand topologies were prepared by employing the PRODRG server. Every protein complex system was solvated with a simple point charge (SPC) water model. Each system was neutralized by attaching chloride ions, accompanied by energy minimization with the steepest descent method of integration. After minimization, the protein was equilibrated for 10 ns at 300 K in NVT as well as NPT ensemble (35). Finally, MD simulation was performed at a temperature of 300 K for 100 ns under periodic boundary state and the time constant of 1.0 ps for coupling. The constant pressure and temperature (1/atm/300 K) were managed through Berendsen Coupling Algorithm17 with a time constant of 0.2 ps for heat-bath coupling (36). The SHAKE algorithm was used during simulation to maintain the length of the bond involving the hydrogen bond. The free binding energies of the selected complexes were calculated with g_mmpbsa software in GROMACS 4.6.7. The MM-PBSA method was applied to the calculation of the binding free energies (37). It can be calculated via the following equation:

(1) $$\begin{array}{r} \text{Δ}{\text{G}}_{\text{binding}}={\text{G}}_{\text{Complex}}-\left[{\text{G}}_{\text{Protein}}+{\text{G}}_{\text{Ligand}}\right] \end{array}$$

Here, ΔG_binding_ delineates the binding free energy of the protein–ligand complexes; G_Protein_ and G_Ligand_ delineate the overall free energies of the protein and ligand molecule. The generated trajectories of MD simulations were then practiced to construct the graph for root mean square deviation (RMSD), RMSD conformational clustering, and hydrogen bond; “gmx rms,” “gmx cluster,” and “gmx hbond” scripts of GROMACS were employed to interpret the yield trajectory data.

## Results and Discussion

The availability of a high-resolution crystallographic structure of the RdRp–RNA complex has unlocked the pathway for the development of potential antivirals targeting the particular protein of SARS-CoV-2. Many studies around the world have suggested that high intake of foods rich in bioactive molecules has beneficial impact on human health and may mitigate the possibility of various human ailments, such as diabetes, cancer, Alzheimer's disease, cataracts, stroke, and age-related functional disorders (38). Bioactive molecules are rich in structural diversity and provide a large area of chemical space for the exploration of possible target sites. In our previous study, the bioactive molecules Oolonghomobisflavan-A, Theasinensin-D, and Theaflavin-3-O-gallate of tea showed better binding than the FDA-approved drugs to the active site of the main protease of SARS-CoV-2 (24). Herein, we screened a dataset of bioactive molecules from tea to check and compare their affinity toward the active site of the RdRp–RNA complex of SARS-CoV-2. The top three tea bioactive molecules screened in this study were epicatechin-3,5-di-O-gallate, epigallocatechin-3,4-di-O-gallate, and epigallocatechin-3,5-di-O-gallate.

### Molecular Docking

**Figure 1:**
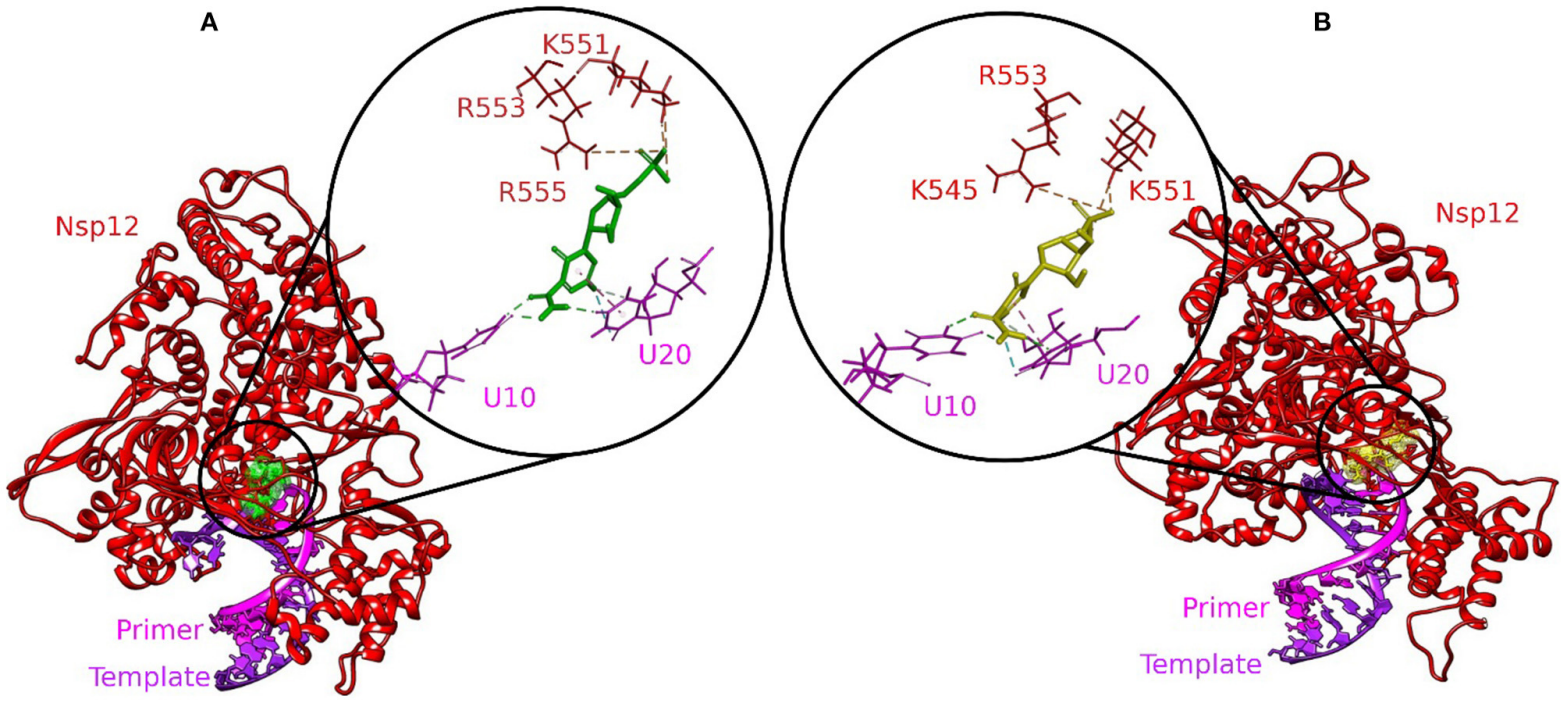
Molecular docking interaction poses of **(A)** Remdesivir and **(B)** Favipiravir with the active site of the RNA–RdRp complex of SARS-CoV-2. The color-coding scheme is as follows: H-bonds (green), pi-alkyl/alkyl (pink), pi-sulfur (golden yellow), and pi-lone pair (limon).

Molecular docking is an exemplary tool to identify the intermolecular framework of ligand–protein, protein–nucleic acid, and protein–protein complexes. The RdRp–RNA complex of SARS-CoV-2 was docked with a set of bioactive molecules from tea and FDA-approved repurposed drugs Remdesivir and Favipiravir. All the molecules were ranked according to their binding scores generated by the CDOCKER protocol of Discovery Studio (Supplementary Table 1). The binding poses of Remdesivir and Favipiravir within the active site of the RdRp–RNA complex were shown in Figure 1. Remdesivir binds to the active site by interacting with the residues of RdRp and RNA nucleotides of both the primer and template strand. The Uracil at position 20 of the primer strand is bound to Remdesivir by two hydrogen bonds and Pi-alkyl interactions. Remdesivir also formed hydrogen bonds with Uracil at position 10 of the template strand. Additionally, Remdesivir was stabilized in the binding site by a Pi-Sulfur interaction with residues Arg555, Lys551, and Arg553 of RdRp. Furthermore, residues Val557, Lys545, Asp760, Cys622, Asp623, Thr680, Ser759, Asn691, Thr687, and Ala688 of RdRp were involved in van der Waals interactions with Remdesivir. Favipiravir occupied the central pocket of the RdRp–RNA complex and formed a hydrogen bond with Uracil at position 20 of the primer RNA strand. Two hydrogen bonds were formed between Favipiravir and Uracil at position 10 of the template RNA strand. Residues Lys545 and Lys551 were involved in Pi-Sulfur interaction with Favipiravir. Residues Arg555, Val557, Ser6682, Asp761, Asp760, and Adenine at position 11 of the template strand were involved in van der Waals interactions.

**Figure 2:**
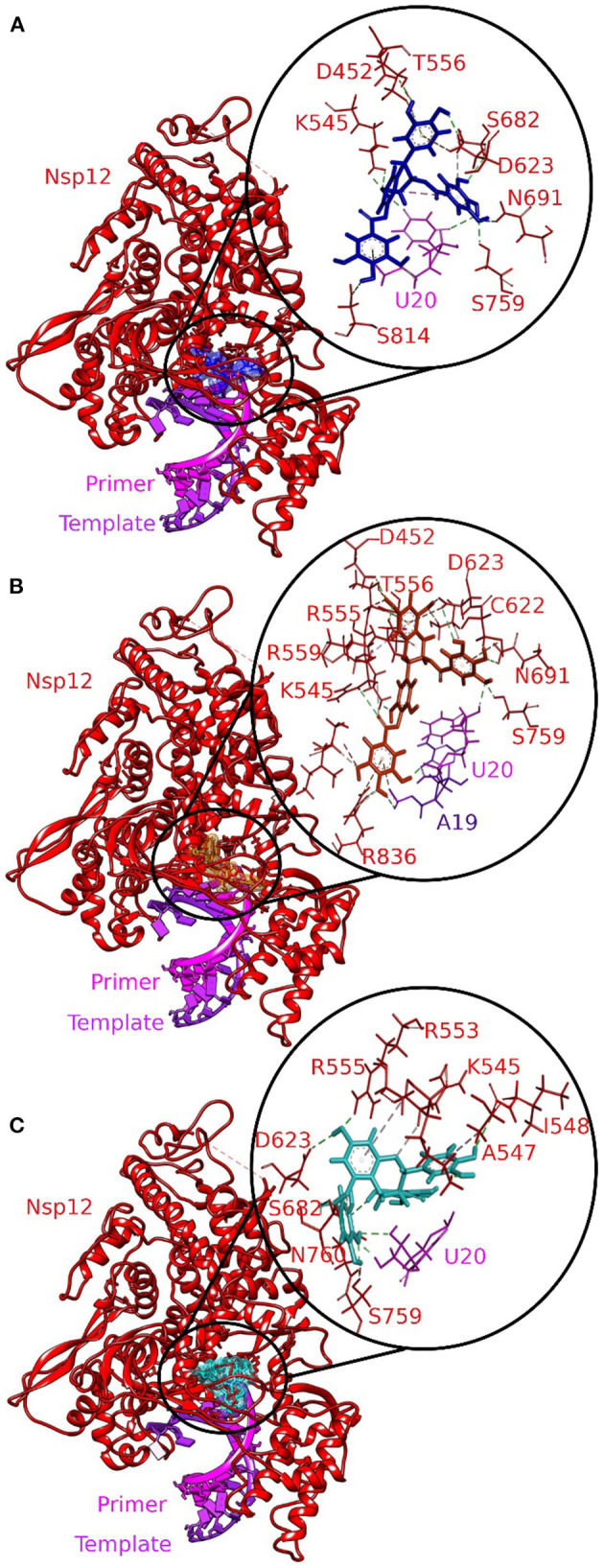
Molecular docking interaction poses of bioactive molecules from tea with the active site of the RNA–RdRp complex of SARS-CoV-2. **(A)** Epicatechin-3,5-di-O-gallate, **(B)** epigallocatechin-3,5-di-O-gallate, and **(C)** epigallocatechin-3,4-di-O-gallate. The color-coding scheme is as follows: H-bonds (green), pi-alkyl/alkyl (pink), pi-sulfur (golden yellow), and pi-lone pair (limon).

Three molecules from tea displayed stronger binding with the active site of the RdRp–RNA complex in terms of CDOCKER interaction energy (Table 1). The docking poses with the most favorable interaction patterns are shown in Figure 2. The molecule epicatechin-3,5-di-O-gallate formed three hydrogen bonds and a Pi-anion interaction with the Uracil at position 20 of the primer RNA strand. The molecule was further stabilized within the active pocket by eight hydrogen bonds with residues Lys545, Asp623, Asp425, Asn691, Ser759, Ser682, and Ser814. Residues Asp623 and Thr556 were involved in the formation of Pi-anion and Pi-Lone Pair interactions, respectively. Many other residues of protein RdRp along with the Adenine and Uracil of the template RNA strand at positions 11 and 10 showed van der Waals interactions. The second molecule from tea, epigallocatechin-3,5-di-O-gallate, formed two hydrogen bonds with Uracil at position 20 of primer RNA. It interacted with residues Ser682, Asp623, Arg553, Arg55, Lys545, Ile548, Asp760, and Ser759 via nine hydrogen bonds. Residues Arg555 and Ala547 were also involved in Pi-alkyl interactions. Furthermore, the binding of epigallocatechin-3,5-di-O-gallate to the active pocket of the RdRp–RNA complex was enhanced by van der Waals interactions by residues Cys622, Thr680, Ser682, Arg624, Val557, Ser549, Ser814, Asp761, Ala688, Thr687, and Asn691. The molecule epigallocatechin-3,4-di-O-gallate also showed higher binding potential than Favipiravir and Remdesivir at the active site of the RdRp–RNA complex. The primer strand Uracil at position 10 interacted via two hydrogen bonds, while the Adenine at position 20 of the same RNA strand formed one hydrogen bond with epigallocatechin-3,4-di-O-gallate. The residues Arg836, Arg555, Ser759, Asn691, Asp623, Ser682, Asp452, Arg553, and Lys545 of the RdRp protein showed 10 hydrogen bonds with epigallocatechin-3,4-di-O-gallate. Moreover, three Pi-anion interactions (Arg836, Cys622, Asp623), two Pi-alkyl interactions (Arg555 and Val557), and a Pi Lone Pair interaction were also observed at the active site.

**Table 1:** Selected tea bioactive molecules and FDA-approved drugs based on -CDOCKER interaction energy.

**Molecules**	**-CDOCKER interaction energy**
Epigallocatechin-3,5-di-O-gallate	79.4086
Epicatechin-3,5-di-O-gallate	78.0801
Epigallocatechin-3,4-di-O-gallate	74.4848
Favipiravir	74.1136
Remdesivir	33.9374

Remdesivir is a nucleotide analog that occupied the central position of the catalytic active site and formed a covalent bond with the primer RNA strand, and terminates replication by non-obligate RNA chain termination (12). However, studies contrary to these results showed the addition of more nucleotides to the RNA strand even after the incorporation of Remdesivir resulting in delayed chain termination (20, 39). The catalytic center of RdRp protein is composed of residues Ser759, Asp760, and Asp761. This site is conserved in most viral RdRps (12, 29). Residues Lys545, Arg553, and Arg555 contribute to the formation of the NTP entry channel (12). The FDA-approved drugs Remdesivir and Favipiravir formed weaker van der Waals and Pi-Sulfur interaction with the residues of the catalytic center and the NTP entry channel. However, all the three selected tea molecules formed stronger hydrogen bonds with most of these residues. Residues Asn691, Ser682, and Asp623 impart specificity to RNA replication over DNA strand by recognizing the RNA specific 2′-OH group (40). Our selected molecules from tea formed stronger hydrogen bonds with residues Asn691, Ser682, and Asp623 as compared to weaker van der Waals interactions formed by Remdesivir and Favipiravir. Stronger interactions with RNA recognition residues and other residues involved in the formation of the catalytic center and the NTP entry channel would ensure the destabilization of the incoming RNA in the active site and hence halt/meddle with the process of viral replication. Furthermore, MD simulations were conducted to substantiate the molecular docking results and explore the dynamics of ligand–protein interactions at the catalytic site of the RdRp–RNA complex.

### Molecular Dynamics Simulations

The basic understanding of how biological macromolecules function requires an awareness of molecular structure and dynamics (41). MD simulations establish fundamental links based on experimental and theoretical evidences between structure and dynamics, allowing the investigation of conformational energy landscape available to biological macromolecules (42, 43). In our previous studies, we showed the potential of bioactive tea molecules to inhibit the Mpro (24) and nsp15 (25) of SARS-CoV-2. Moreover, in a different study, we demonstrated the inhibitory potential of acridinedione analogs to inhibit the Mpro of SARS-CoV-2 (44). A recent study employing MD simulations suggested potential molecules to target the RdRp of SARS-CoV-2 (45). The RdRp–RNA complexes with Remdesivir, Favipiravir, and three selected bioactive molecules of tea were subjected to explicit MD simulations. The MD results were analyzed by using different multiscale computational methods.

### Structural Stability of RdRp–Ligand–RNA Complexes

**Figure 3:**
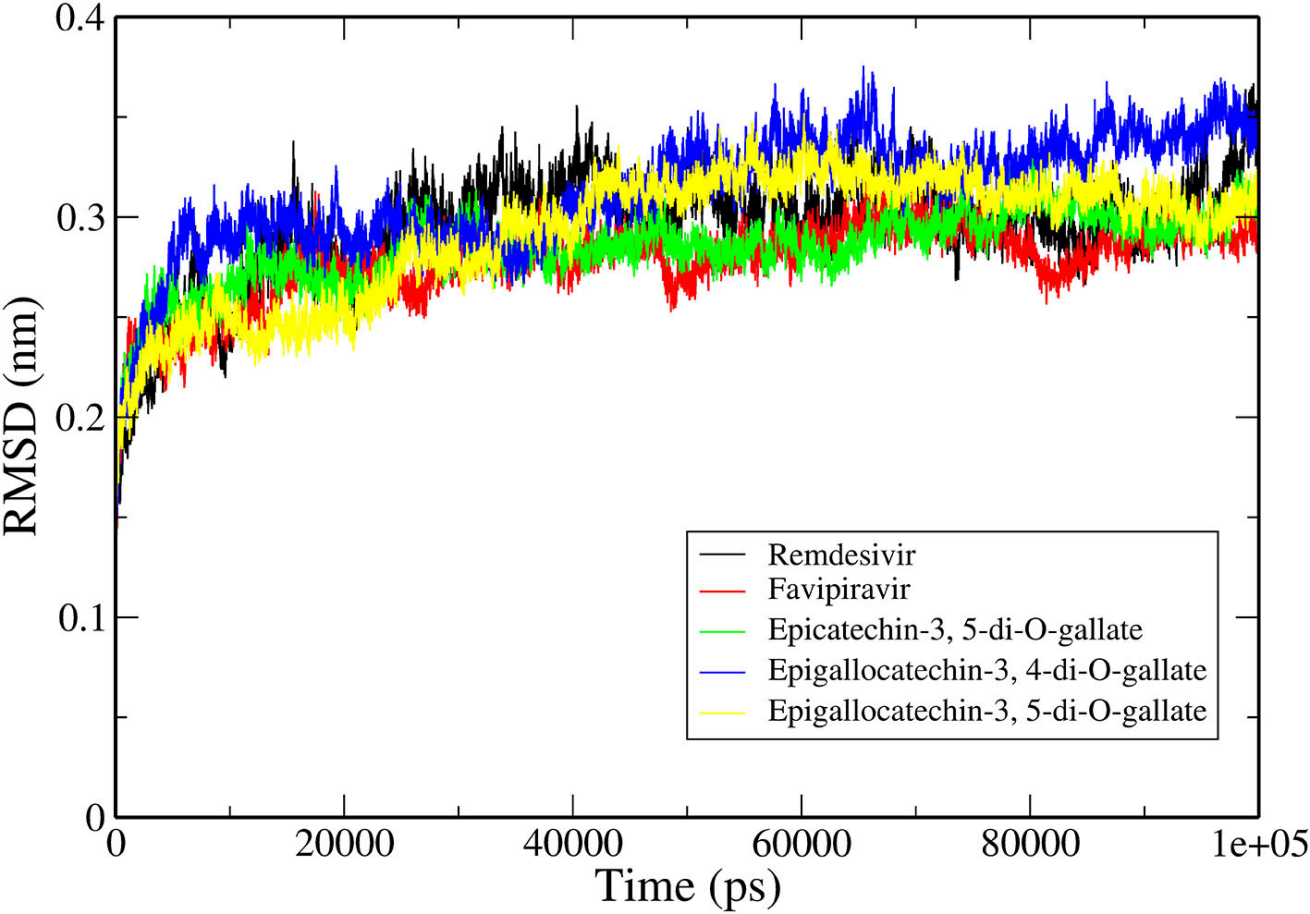
Backbone RMSDs are shown as a function of time for the RNA–RdRp complex of SARS-CoV-2 and ligands.

The RMSD is a classical technique for the analysis of MD results. It is a popular measure for analyzing the structural stability of protein structures. The RMSDs of all the C-α atoms of selected protein complexes were calculated, as depicted in Figure 3. All the protein structures deviated < 0.35 nm during the simulation. The RdRp–RNA complex with Remdesivir had the highest deviation with an average RMSD value of ~0.32 nm. The complex with Favipiravir deviated at a lower trajectory than Remdesivir after 25 ns until the end of the simulation. All the three RdRp–RNA complexes having selected tea molecules (epicatechin-3,5-di-O-gallate, epigallocatechin-3,4-di-O-gallate, and epigallocatechin-3,5-di-O-gallate) showed relatively lower deviations than Remdesivir. The average RMSD values of epicatechin-3,5-di-O-gallate and epigallocatechin-3,5-di-O-gallate were 0.27 and 0.29 nm, respectively. After initial deviations from the starting structure for the first 25 ns, the RMSD trajectories of the simulated complexes stabilized and showed convergence in the second half of the simulation. These results indicated that the structural stability of RdRp–RNA complexes with bioactive molecules of tea was comparable to that of Favipiravir and was more stable than Remdesivir. Additionally, lower RMSD values also showed that all the simulated structures were able to reproduce correct binding poses as generated by molecular docking studies.

### Dynamics of Simulated Complexes

**Figure 4:**
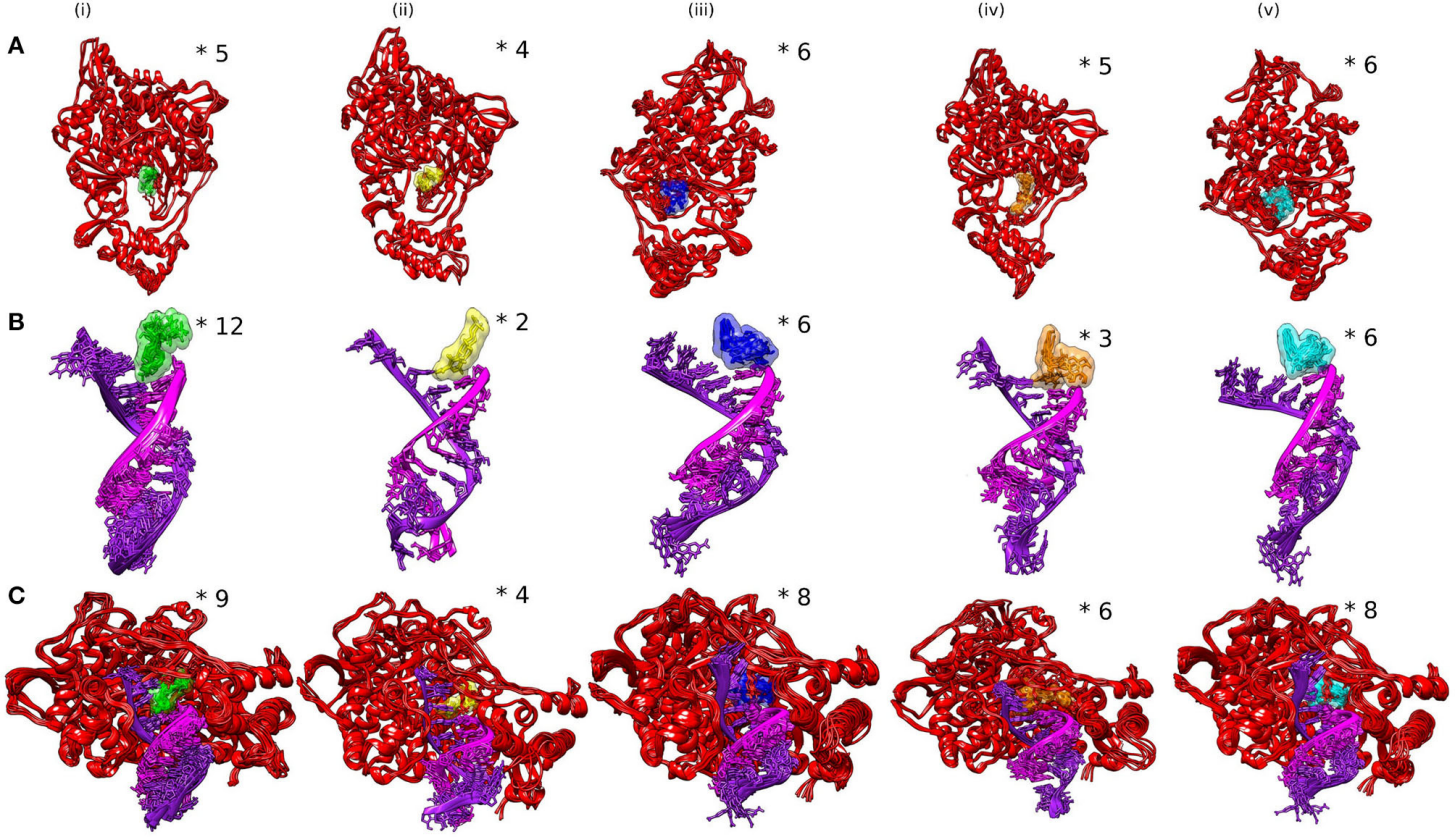
Analysis of SARS-CoV-2 complexes shown as clusters of **(A)** RdRp–ligands, **(B)** RdRp–RNA, and **(C)** RdRp–RNA–ligands. Numbers of clusters are represented by asterisk.

The ensemble clustering of simulation data is a conclusive and effectual method of analyzing the structural flexibility of concerned protein systems (46). The MD trajectories of all the selected complexes for 5 ns (45–50 ns) of the simulation period were extracted and subjected to clustering analysis. The clustering was done on three different combinations of the receptor and the ligand to explore the dynamics of ligand interactions with individual receptors and the whole protein (RdRp)–nucleic acid (active site RNA) complex. The RMSD clustering results are shown in Figure 4. In RdRp–ligand complexes, Favipiravir formed the least number of clusters with an average RMSD of 0.130 nm, while Remdesivir and epigallocatechin-3,4-di-O-gallate formed five clusters each with an average RMSD of 0.131 and 0.130 nm, respectively. In RNA–ligand complexes, Favipiravir showed only two clusters with an average RMSD of 0.109 nm. Among the bioactive molecules of tea, epigallocatechin-3,4-di-O-gallate showed only three clusters, while the rest of the two molecules formed six clusters each while interacting with RNA. Remdesivir formed 12 clusters with an average RMSD of 0.160 nm. Similarly, in protein–RNA–ligand complexes, Remdesivir formed the most number of clusters (nine clusters) followed by epicatechin-3,5-di-O-gallate (eight clusters), epigallocatechin-3,5-di-O-gallate (eight clusters), epigallocatechin-3,4-di-O-gallate (six clusters), and Favipiravir (four clusters). The average RMSD of all the clusters was below 0.140 nm. These results showed that the selected bioactive tea molecules were more stable than Remdesivir and almost comparable to Favipiravir in clustering analysis. Furthermore, to visualize the effect of structural fluctuations on intermolecular interactions, we analyzed the hydrogen bond formations between RdRp–ligand and RNA–ligand complexes.

### Analysis of Intermolecular Hydrogen Bonds

**Figure 5:**
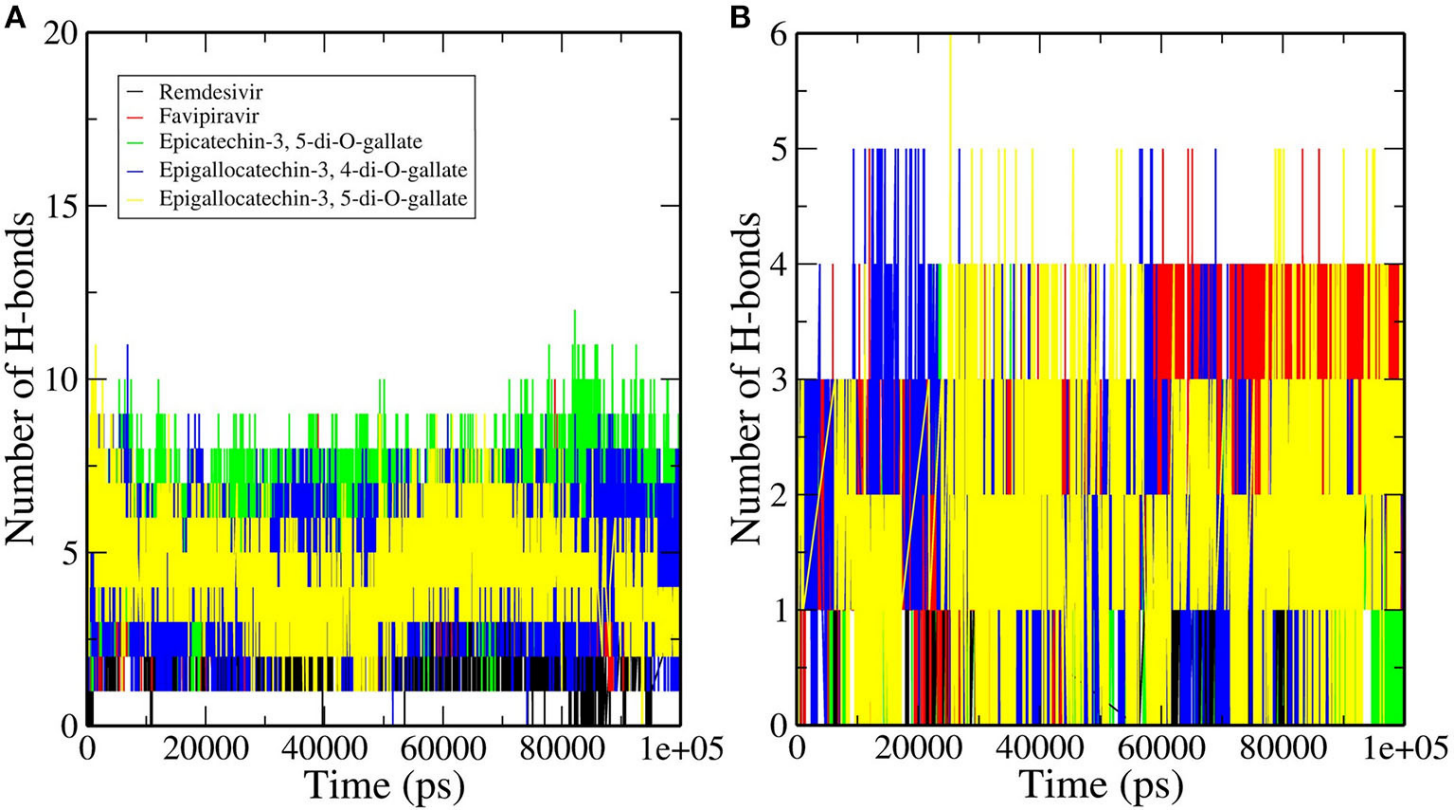
Hydrogen bond profiles of the RNA–RdRp complex of SARS-CoV-2 having **(A)** RdRp and **(B)** RNA.

Hydrogen bonds are commonly considered as mediators of protein–ligand binding and also promote the binding affinity of a ligand by displacing the water molecules bound to protein into the bulk solvent. We calculated the number of hydrogen bonds formed by the selected bioactive molecules of tea and standard drugs with both the RdRp and RNA of SARS-CoV-2. Remdesivir and Favipiravir formed an average of four and five hydrogen bonds, respectively, with the RdRp of SARS-CoV-2. Epicatechin-3,5-di-O-gallate formed the most number of hydrogen bonds during the simulation with the residues of the RdRp of SARS-CoV-2. The average number of hydrogen bonds formed during the simulation by epicatechin-3,5-di-O-gallate was 7, with few conformations formed up to 10 hydrogen bonds (Figure 5A). Epigallocatechin-3,4-di-O-gallate stabilized in the binding pocket by forming an average of six hydrogen bonds with the RdRp of SARS-CoV-2. The third selected molecule epigallocatechin-3,5-di-O-gallate for the first 25 ns showed an average of five hydrogen bonds, while for the next 25 ns, the average number of hydrogen bonds was five with RdRp. Similarly, epicatechin-3,5-di-O-gallate, epigallocatechin-3,4-di-O-gallate, and epigallocatechin-3,5-di-O-gallate formed more hydrogen bonds with RNA at the active site of RdRp than Remdesivir and Favipiravir. The average number of hydrogen bonds formed between epicatechin-3,5-di-O-gallate and RNA was two, while the highest number of hydrogen bonds was four. Both epigallocatechin-3,4-di-O-gallate and epigallocatechin-3,5-di-O-gallate showed up to five hydrogen bonds with the active site RNA (Figure 5B). The standard drugs Remdesivir and Favipiravir were able to form an average of one and two hydrogen bonds, respectively, with the active site RNA. All the three selected bioactive molecules of tea formed a greater number of hydrogen bonds within the active site of the RdRp of SARS-CoV-2 than Remdesivir and Favipiravir throughout the simulation period. These results were further confirmed by extracting RdRp–ligand–RNA complex trajectories at different time intervals and visualizing the stability of selected molecules in the active site of RdRp (Supplementary Figure 1). The bioactive molecules of tea were tightly bound to the active site of RdRp by forming a greater number of hydrogen bonds and other non-covalent interactions than Remdesivir and Favipiravir. Furthermore, an efficient and reliable method of calculating the binding free energy and its contributors was employed to compare bioactive tea molecules with Remdesivir and Favipiravir.

### The Molecular Mechanics Poisson–Boltzmann Surface Area (MM-PBSA) Analysis

**Figure 6:**
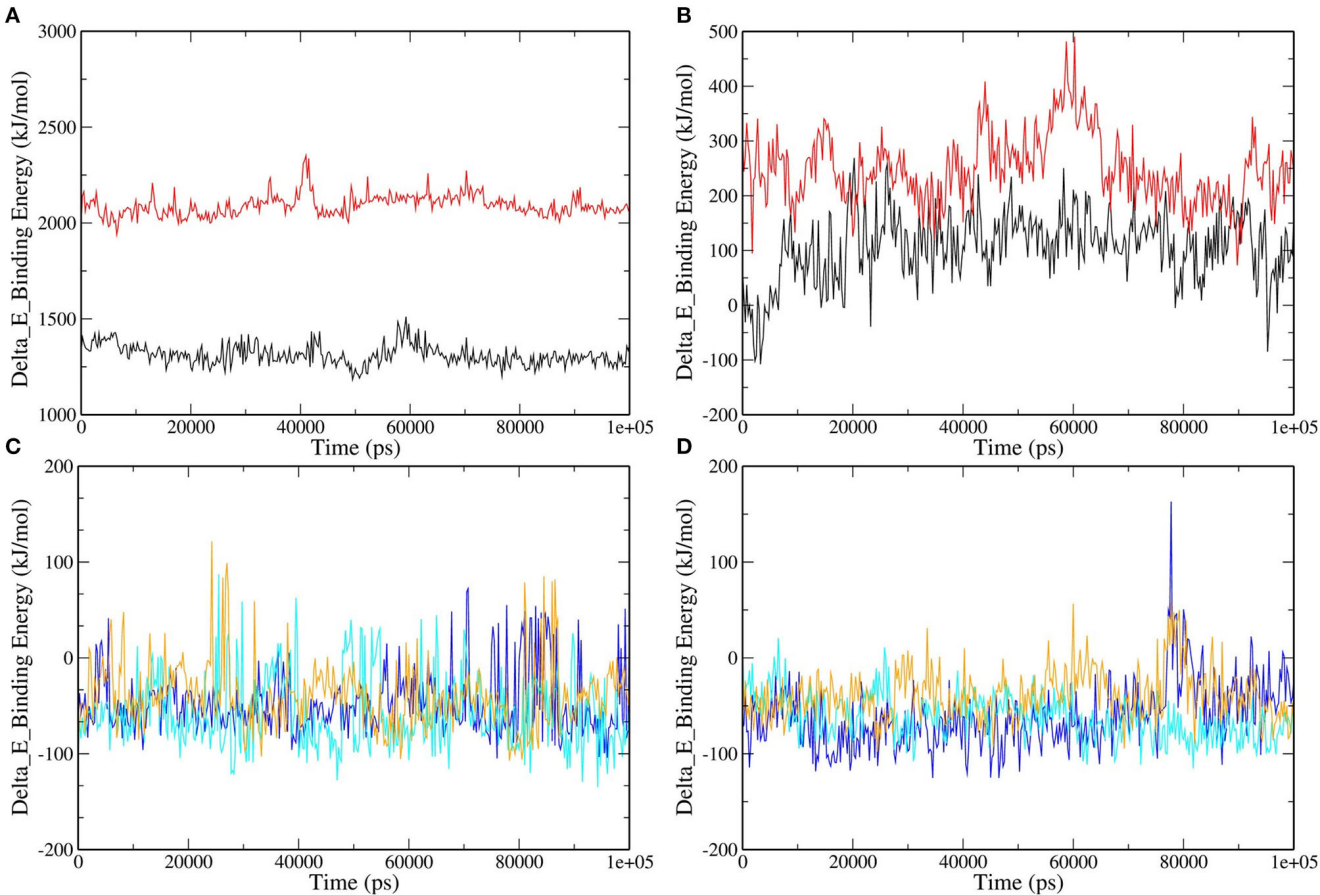
Binding free energy represented graphically for RdRp protein and color scheme as follows: **(A)** RNA–Remdesivir (black), RNA–Favipiravir (red). **(B)** RdRp–Remdesivir (black), RdRp–Favipiravir (red). **(C)** RNA–epicatechin-3,5-di-O-gallate (blue), RNA–epigallocatechin-3,5-di-O-gallate (cyan), RNA–epigallocatechin-3,4-di-O-gallate (orange). **(D)** RdRp–epicatechin-3,5-di-O-gallate (blue), RdRp–epigallocatechin-3,5-di-O-gallate (cyan), RNA–epigallocatechin-3,4-di-O-gallate (orange).

MD simulations present a glimpse of a ligand's stability in the binding region of a concerned protein. By implementing MM-PBSA calculations, we assessed the binding free energy of selected RdRp–ligand complexes. The observation was made with the extraction of the RdRp–ligand complex scripts from MD simulations. The binding free energy can acceptably illustrate the durability of the linking ligand receptor, which is an integral aspect of drug development. The binding energies of the ligands with RdRp and RNA were compared. Remdesivir and Favipiravir showed positive binding energy with both RdRp and RNA. Moreover, the binding free energy was decomposed into electrostatic, SASA, van der Waals, and polar solvation energies. The lesser the binding energy, the more reliable the ligand–protein binding. The favorable contribution of SASA, electrostatic, and van der Waals energies was devoted to the binding of RdRp and RNA with our selected molecules from tea. In contrast, electrostatic energy was positive for standard molecules in RNA but favorable in RdRp, as shown in Figure 6 and Table 2. Approximately all atoms inside a macromolecule convey a partial charge, and thus, molecules striving for molecular classification interact via electrostatic interactions. It is believed that these interactions assist two leading roles: to control the molecules toward their binding style and to generate unique interactions within the active site (47). It is assumed that the positive values of the electrostatic associations destabilize the interaction and thus diminish the affinity. By contrast, in binding free energy, the polar solvation energy participated generously to increase the total energy. Van der Waals energy's augmentation to the overall binding free energy was higher upon the electrostatic contribution energy. The higher (–ve) binding energy is responsible for potential binding. These results bestow higher binding energy for all the selected tea molecules as compared to Remdesivir and Favipiravir.

**Table 2:** Binding free energy (MM-PBSA) calculations for selected complexes.

**RNA–RdRp–Ligand Complexes**	**ΔE _**binding (kJ/mol)**_**	**ΔE _**polar solvation (kJ/mol)**_**	**SASA _**(kJ/mol)**_**	**ΔE _**Electrostatic (kJ/mol)**_**	**ΔE _**Van der Waal (kJ/mol)**_**
RdRp-Epicatechin-3,5-di-O-gallate	−61.461	209.138	−19.313	−87.878	−163.408
RdRp-Epigallocatechin-3,5-di-O-gallate	−38.515	280.167	−18.915	−139.544	−160.222
RdRp-Epigallocatechin-3,4-di-O-gallate	−62.278	249.433	−22.516	−110.399	−178.796
RdRp-Remdesivir	107.346	437.201	−12.356	−248.406	−69.093
RdRp-Favipiravir	248.378	977.029	−12.653	−657.208	−58.790
RNA-Epicatechin-3,5-di-O-gallate	−47.148	66.063	−7.478	−41.142	−64.592
RNA-Epigallocatechin-3,5-di-O-gallate	−39.857	58.538	−7.632	−27.821	−62.942
RNA-Epigallocatechin-3,4-di-O-gallate	−56.695	99.860	−10.168	−55.757	−90.630
RNA-Favipiravir	2091.712	−18.286	−6.780	2168.615	−51.572
RNA-Remdesivir	1311.862	−30.025	−7.045	1387.624	−40.383

## Conclusion

The RdRp is an attractive target for the development of specific inhibitors for SARS-CoV-2. The FDA-approved drugs Remdesivir and Favipiravir showed promising results in curing COVID-19 patients. In this study, a dataset of bioactive molecules from tea was screened to analyze its interaction profiles within the active site of the RdRp–RNA complex. The molecules epicatechin-3,5-di-O-gallate, epigallocatechin-3,4-di-O-gallate, and epigallocatechin-3,5-di-O-gallate formed stronger hydrogen bonds with the key residues involved in the recognition of RNA for replication, the catalytic center, and the NTP entry channel. These residues showed weaker van der Waals interactions with Remdesivir and Favipiravir. Both the selected molecules also showed the most favorable binding energies during robust MD simulations than the standard drug molecules. The bioactive molecules of tea also target the Mpro and nsp15 of SARS-CoV-2 as shown by our previous reports. The results our previous study along with the present findings disclose the ability of bioactive molecules of tea to target multiple proteins of SARS-CoV-2 (Mpro, nsp15, and RdRp). These bioactive molecules could be quickly made available in formulations along with other antiviral therapies to rapidly cure COVID-19 patients. These *in silico* results, however, require validation by experimental studies.

## Data Availability Statement

The original contributions presented in the study are included in the article/Supplementary Material, further inquiries can be directed to the corresponding author/s.

## Author Contributions

RP conceived of and designed the study. VKB, RS, JS, and VR analyzed and interpreted the data. RS, VKB, VR, RP, and SK critically revised it for important intellectual content. All authors gave final approval of the version to be published.

## Conflict of Interest

The authors declare that the research was conducted in the absence of any commercial or financial relationships that could be construed as a potential conflict of interest.
