# Supplementary material for: Bioactive Molecules of Tea as Potential Inhibitors for RNA-Dependent RNA Polymerase of SARS-CoV-2
Source: Front Med (Lausanne). 2021 May 31;8:684020. doi: 10.3389/fmed.2021.684020 (PMC8200525; doi:10.3389/fmed.2021.684020)
Supplement: Supplementary file 1 [file Data_Sheet_1.PDF]

Supplementary Materials for

Bioactive molecules of the Tea as potential inhibitors of the RNA-dependent RNA polymerase of SARS-CoV-2

Vijay Kumar Bhardwaj[1,2,3], Rahul Singh[1,2], Jatin Sharma[1,2,], Vidya Rajendran[2], Rituraj Purohit[*1, 2, 3], and Sanjay Kumar[2]

[1]Structural Bioinformatics Lab, CSIR-Institute of Himalayan Bioresource Technology (CSIR-IHBT), Palampur, HP, 176061, India

[2]Biotechnology division, CSIR-IHBT, Palampur, HP, 176061, India

[3]Academy of Scientific & Innovative Research (AcSIR), CSIR-IHBT Campus, Palampur, HP, 176061, India

*Author for correspondence (rituraj@ihbt.res.in)

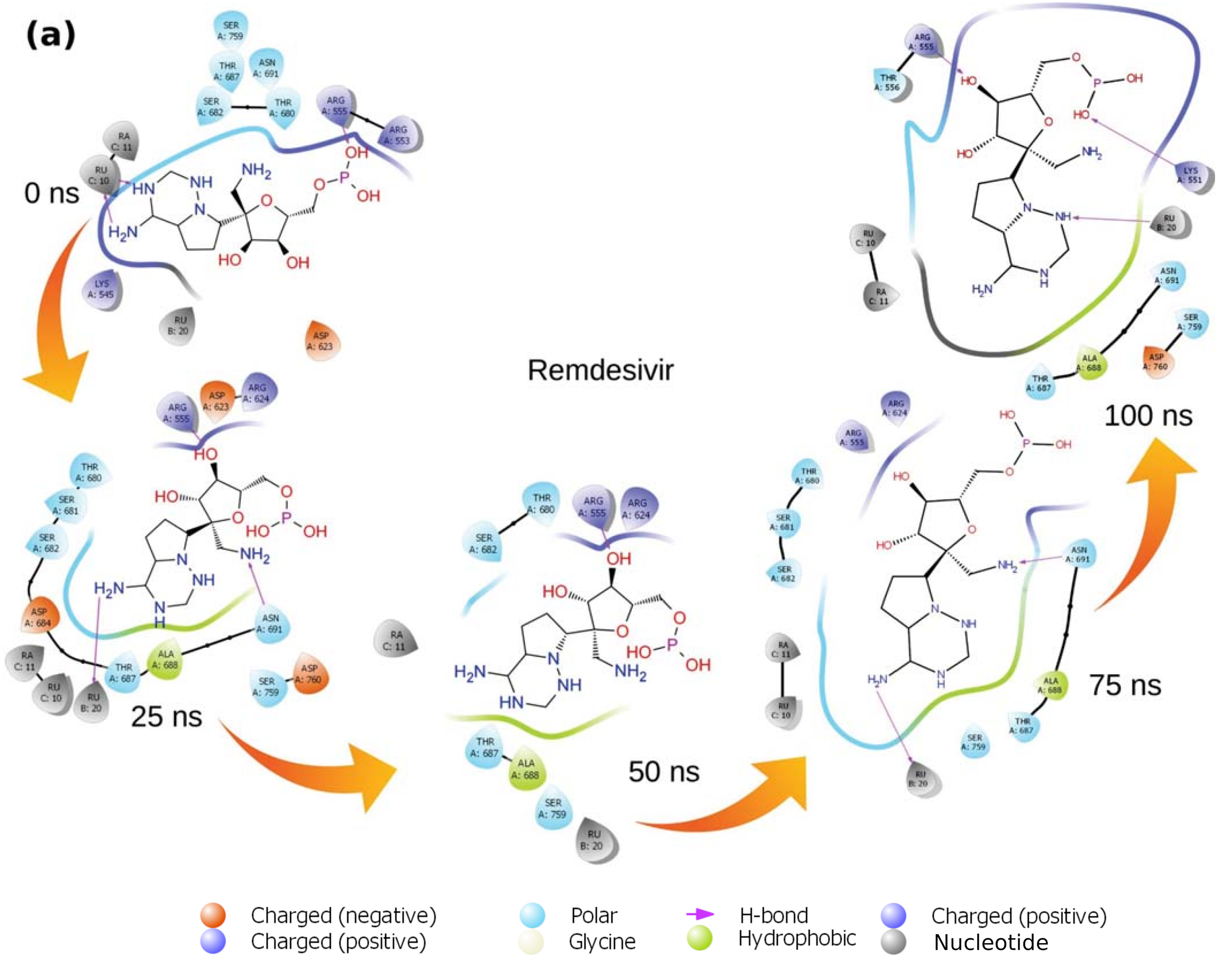
(a)
0 ns
25 ns
Remdesivir
50 ns
75 ns
100 ns
Charged (negative)
Charged (positive)
Polar
Glycine
H-bond
Hydrophobic
Charged (positive)
Nucleotide

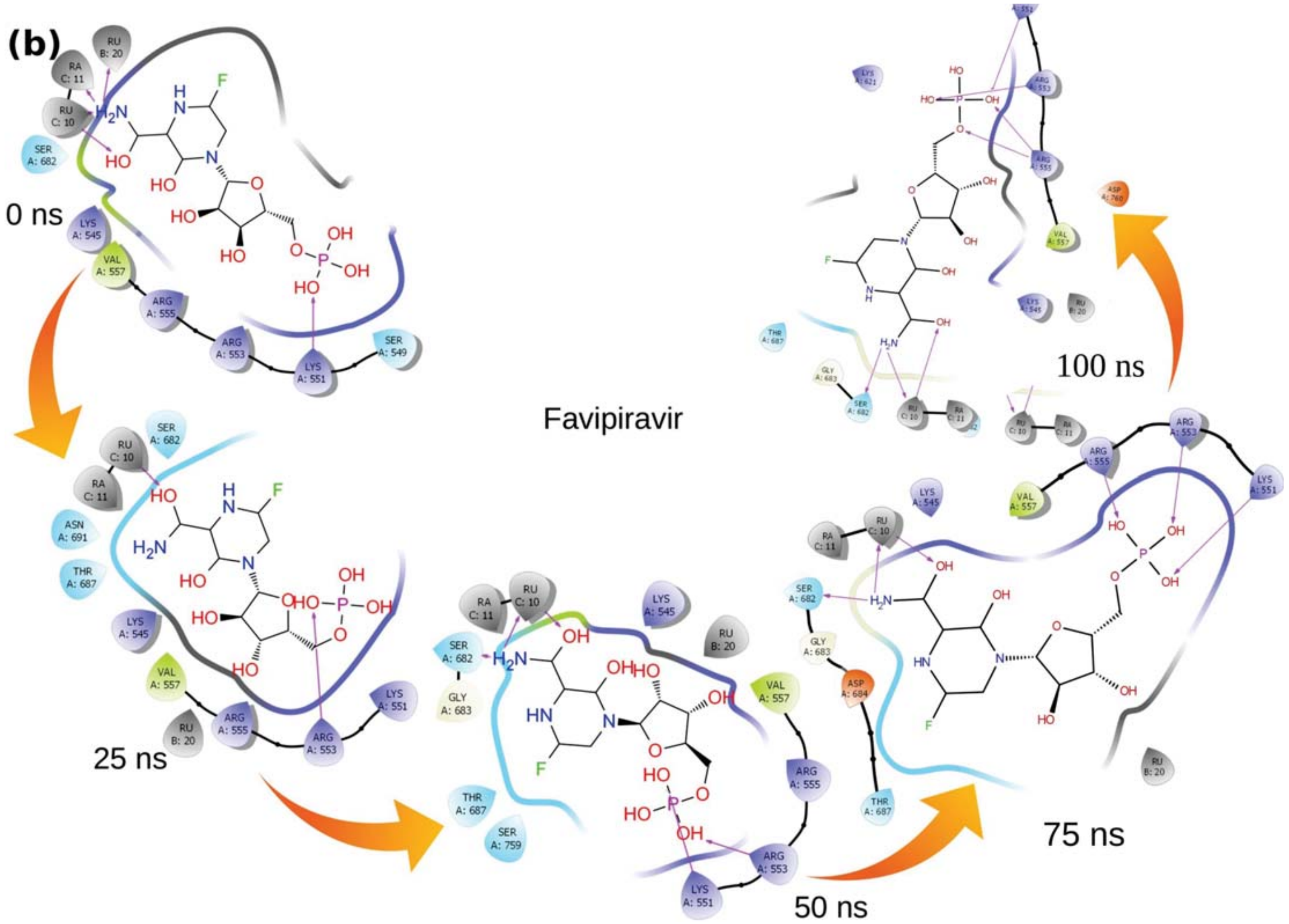
(b)
Favipiravir
0 ns
25 ns
50 ns
75 ns
100 ns

(c)

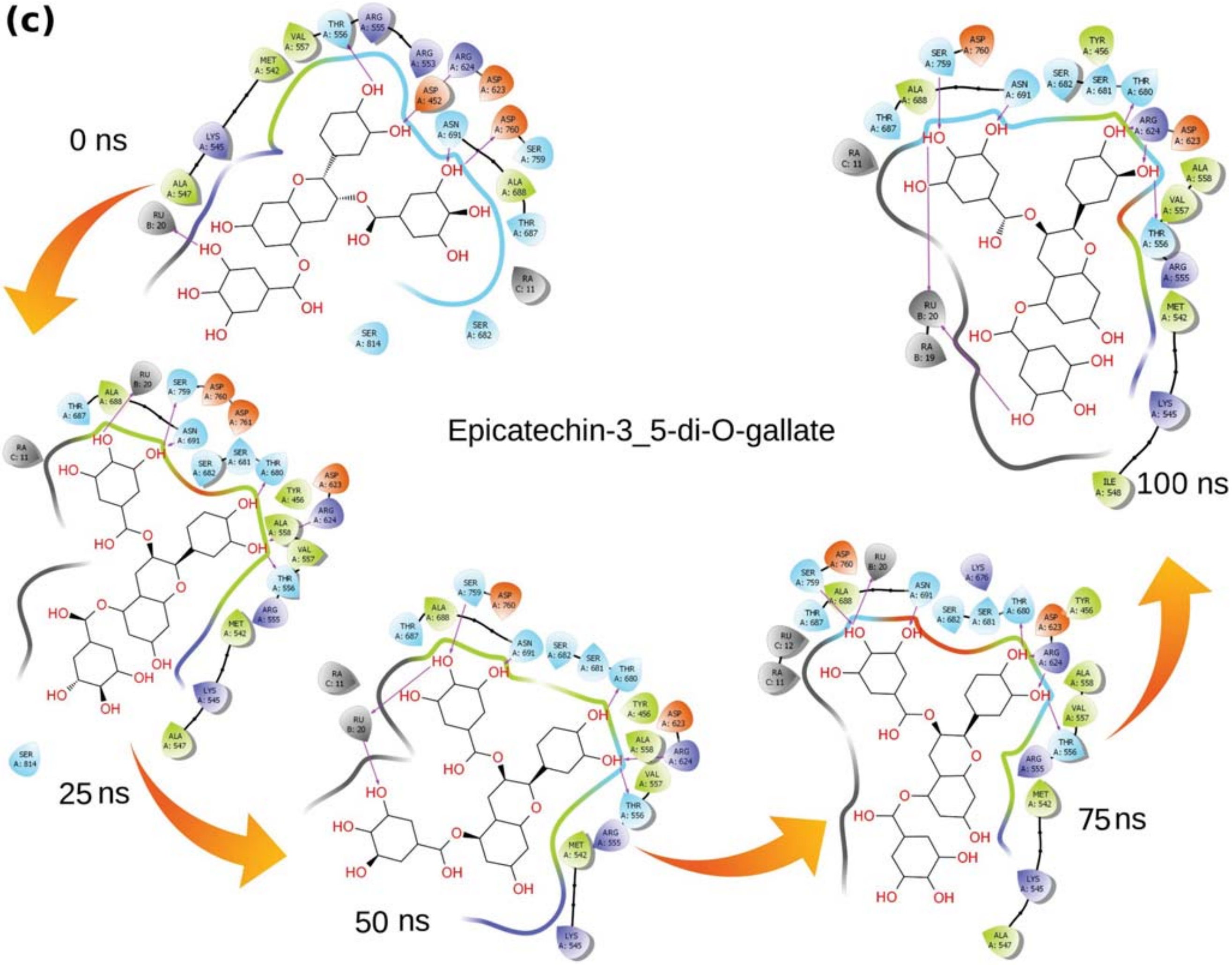

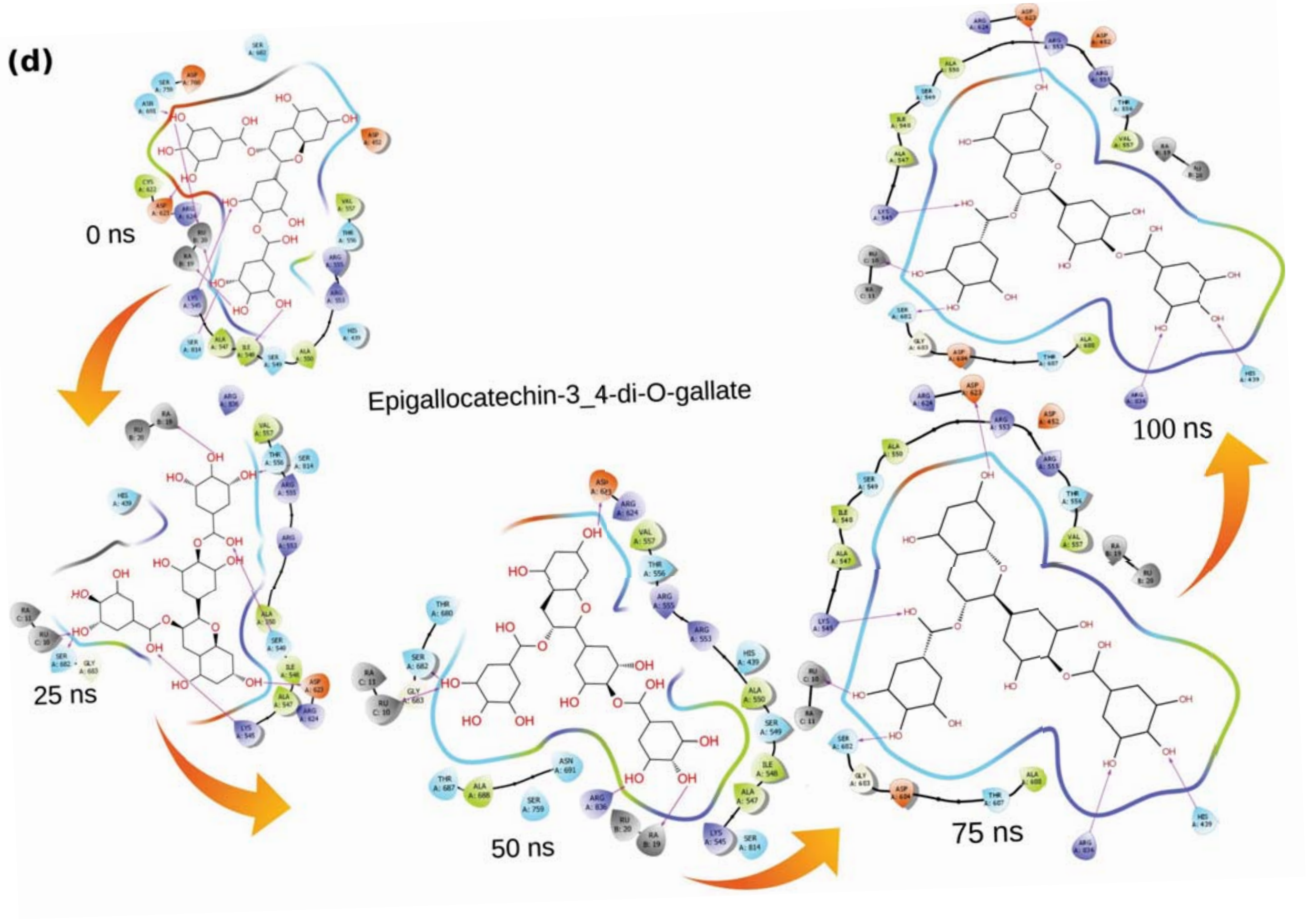
(d)
0 ns
Epigallocatechin-3_4-di-O-gallate
25 ns
50 ns
75 ns
100 ns

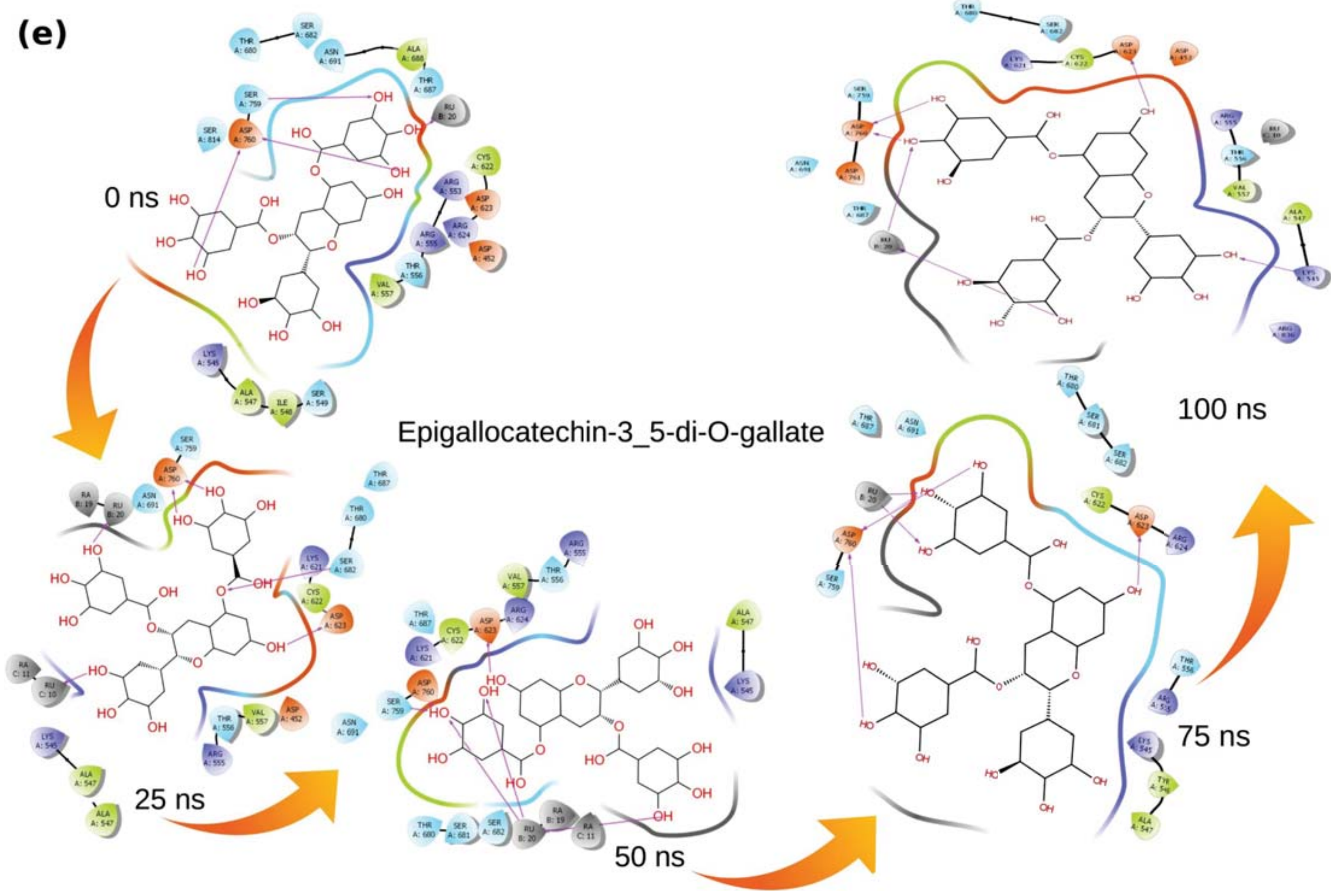

Figure S1. 2D interactions showing hydrogen bonds (pink arrow) at different time period of MD simulations in the binding pocket of the RNA-RdRp complexes of SARS-CoV-2 visualized by academic version of Maestro.

Table S1. CDOCKER interaction energies of selected 64 bioactive molecules and 2 FDA approved drugs.

S. No.	Molecules	-CDOCKER Interaction Energy
1.	Epigallocatechin-3,5-di-O-gallate	79.41
2.	Epicatechin-3,5-di-O-gallate	78.08
3.	Epigallocatechin-3,4-di-O-gallate	74.48
4.	Favipiravir	74.11
5.	Theaflavin-3'-O-gallate	66.83
6.	Epicatechin gallate	63.41
7.	Epigallocatechin gallate	61.11
8.	Epitheaflavic Acid 3'-Gallate	56.3
9.	Epiafzelechin 3-O-gallate	55.88
10.	Epigallocatechin 3-O-p-coumarate	52.36
11.	Theasinensin E	48.18
12.	Myricetin	43.91
13.	Quercetin	41.37
14.	Theaflavin	40.85
15.	Chlorogenic acid	40.73
16.	Proanthocyanidin	39.75
17.	Epicatechin	39.02
18.	Gallocatechin	38.39
19.	Catechin	37.04
20.	Epigallocatechin	36.05
21.	Theanine	35.68
22.	Vitexin	35.19
23.	Kaempferol	35.13
24.	Apigenin	34.26
25.	Remdesivir	33.94
26.	Coumaroyl quinic acid	31.77
27.	Isoquercetin	31.38
28.	Theaflavic acid	31.11
29.	Rutin	29.02
30.	Succinic acid	28.67
31.	Isovitexin	27.52
32.	Myricitrin	24.43
33.	Theobromine	23.94
34.	Hexanal	23.46

35.	Methyl salicylate	23.33
36.	Vicenin 2	23.25
37.	Saponarin	21.54
38.	Methylxanthine	21.54
39.	Phenylacetaldehyde	21.17
40.	Phenylethanol	18.74
41.	Xanthine	18.65
42.	Oxalic acid	18.2
43.	trans-2-Hexenal	17.96
44.	Caffeine	17.45
45.	Theophylline	17.3
46.	Benzaldehyde	14.25
47.	Diphenylamine	14.06
48.	Ascorbic acid	13.34
49.	Isotheaflavin	10.46
50.	Pheophytin	10.03
51.	Quinic acid	9.55
52.	Kaempferitrin	6.98
53.	cis-3-Hexenol	4.66
54.	Linalool oxide	2.15
55.	Linalool	-3.69
56.	Nerolidol	-16.54
57.	Geraniol	-17.51
58.	Barringtogenol	-64.15
59.	Barrigenol A1	-66.64
60.	Barrigenol R1	-69.54
61.	Camelliagenin	-73.99
62.	Pheophorbide	-98.87
63.	Cryptoxanthin	-470.85
64.	Oolonghomobisflavan A	Pose not generated
65.	Theasinensin D	Pose not generated
66.	Theasinensin F	Pose not generated
67.	Oolonghomobisflavan B	Pose not generated
